# PEST-containing nuclear protein mediates the proliferation, migration, and invasion of human neuroblastoma cells through MAPK and PI3K/AKT/mTOR signaling pathways

**DOI:** 10.1186/s12885-018-4391-9

**Published:** 2018-05-02

**Authors:** Dong-Dong Wu, Ying-Ran Gao, Tao Li, Da-Yong Wang, Dan Lu, Shi-Yu Liu, Ya Hong, Hui-Bin Ning, Jun-Ping Liu, Jia Shang, Jun-Feng Shi, Jian-She Wei, Xin-Ying Ji

**Affiliations:** 1 School of Basic Medical Sciences, Henan University College of Medicine, Kaifeng, 475004 Henan China; 2 Henan Provincial People’s Hospital Affiliated to Henan University, Zhengzhou, 450003 Henan China; 3 Brain Research Laboratory, College of Life Sciences, Henan University, Kaifeng, 475004 Henan China; 4 Nanyang Nanshi Hospital Affiliated to Henan University, Nanyang, 473003 Henan China

**Keywords:** PEST-containing nuclear protein, Neuroblastoma, Angiogenesis, Apoptosis, Signaling pathway

## Abstract

**Background:**

PEST-containing nuclear protein (PCNP), a novel nuclear protein, is involved in cell proliferation and tumorigenesis. However, the precise mechanism of action of PCNP in the process of tumor growth has not yet been fully elucidated.

**Methods:**

ShRNA knockdown and overexpression of PCNP were performed in human neuroblastoma cells. Tumorigenic and metastatic effects of PCNP were examined by tumor growth, migration, and invasion assays in vitro, as well as xenograft tumor assay in vivo.

**Results:**

PCNP over-expression decreased the proliferation, migration, and invasion of human neuroblastoma cells and down-regulation of PCNP showed reverse effects. PCNP over-expression increased protein expressions of cleaved caspase-3, cleaved caspase-8, cleaved caspase-9, and cleaved poly adenosine diphosphate-ribose polymerase, as well as ratios of B-cell lymphoma-2 (Bcl-2)-associated X protein/Bcl-2 and Bcl-2-associated death promoter/B-cell lymphoma-extra large in human neuroblastoma cells, however PCNP knockdown exhibited reverse trends. PCNP over-expression increased phosphorylations of extracellular signal-regulated protein kinase 1/2, p38, c-Jun N-terminal kinase, as well as decreased phosphorylations of phosphatidylinositol 3-kinase (PI3K), Akt, and mammalian target of rapamycin (mTOR), nevertheless PCNP knockdown exhibited opposite effects. Furthermore, PCNP over-expression significantly reduced the growth of human neuroblastoma xenograft tumors by down-regulating angiogenesis, whereas PCNP knockdown markedly promoted the growth of human neuroblastoma xenograft tumors through up-regulation of angiogenesis.

**Conclusions:**

PCNP mediates the proliferation, migration, and invasion of human neuroblastoma cells through mitogen-activated protein kinase and PI3K/AKT/mTOR signaling pathways, implying that PCNP is a therapeutic target for patients with neuroblastoma.

## Background

Neuroblastoma, a pediatric cancer of the developing sympathetic nervous system, is one of the most common solid tumors in infancy and early childhood [1–3]. The tumor emerges in tissues of the sympathetic nervous system, typically in the paraspinal ganglia or adrenal medulla, and thus can present as mass lesions in the chest, neck, pelvis, and abdomen [4]. The prognosis is highly variable and is associated with a number of parameters including tumor stage, age at diagnosis, and grade of differentiation of the tumor [5, 6]. Although the survival of children with neuroblastoma has significantly improved during recent years, patients with advanced-stage disease still show a poor prognosis, despite intensive and advanced treatments, with overall survival probabilities of less than 40% [7–9]. Therefore, it is urgent to clarify the molecular and genetic properties of neuroblastoma that will greatly improve the therapeutic effect of this complex heterogeneous disease [5, 10].

The PEST motif is a peptide sequence which is rich in proline (P), glutamic acid (E), serine (S), and threonine (T) [11–13]. It is well known that the PEST sequence functions as a proteolytic signal to target proteins for degradation via the proteasome pathway or calpain proteolysis [11, 14, 15]. The PEST sequence is considered the unstructured region in a number of protein sequences, possibly serving as a phosphodegron for the recruitment of F-box-containing ubiquitin E3 ligases that result in ubiquitination and degradation [16, 17]. A novel PEST-containing nuclear protein (PCNP) has been identified in the nucleus through database mining. Np95/ICBP90-like RING finger protein (NIRF) is a nuclear protein with a ubiquitin-like domain, a YDG/SRA domain, a PHD finger, and a RING finger. PCNP could interact with NIRF and modulate the transcriptional activity of NIRF [18]. PCNP and NIRF may be involved in the signaling pathway concerned with cell cycle regulation and/or genome stability [19]. In addition, it has been shown that PCNP mRNA can be detected in many types of cancer cells, such as HT-1080 fibrosarcoma cells, HepG2 hepatoma cells, and U-937 myeloid leukemia cells, indicating that PCNP might play an important role in cell proliferation and tumorigenesis [18, 19]. However, the expression level of PCNP in neuroblastoma cells is unknown, and the effect of PCNP on the growth of neuroblastoma cells has not yet been elucidated.

In the present study, we investigated the effects and mechanisms of PCNP on the proliferation, migration, and invasion of human neuroblastoma cells. We further examined the effects of PCNP on tumor growth and angiogenesis in nude mice xenografted with human neuroblastoma.

## Methods

### Cell culture

Human neuroblastoma cell lines SH-SY5Y and SK-N-SH were purchased from CoBioer Biosciences Co., Ltd. (Nanjing, Jiangsu, China) and cultured in RPMI1640 medium supplemented with 10% fetal bovine serum (FBS), 100 U/ml penicillin, and 100 μg/ml streptomycin. Cells were grown in an incubator with a humidified atmosphere of 95% air and 5% CO_2_ at 37 °C.

### Over-expression and knockdown of PCNP

Human PCNP complementary deoxyribonucleic acid (cDNA) (NM_020357) was sub-cloned into the Xho I and Kpn I restrictive sites of GV230 (Genechem, Shanghai, China), validated by sequencing and transfected into tumor cells with Lipofectamine 3000 Transfection Reagent (Life Technologies, Carlsbad, CA, USA). The empty vector (Mock group) or GV230-PCNP construct (PCNP group) was transfected into tumor cells, and stable cell lines were screened by administration of G418 (Solarbio, Shanghai, China). The oligonucleotides encoding short hairpin ribonucleic acid (shRNA) specific for PCNP and their scramble sequences were sub-cloned into the Age I and EcoR 1 restrictive sites of GV248 (Genechem, Shanghai, China). The PCNP shRNA (sh-PCNP group) and scramble shRNA (sh-Scb group) were verified by DNA sequencing and transfected into tumor cells with Lipofectamine 3000 Transfection Reagent. Stable tumor cell lines transfected with shRNAs were screened by administration of puromycin (Solarbio, Shanghai, China). The untransfected tumor cells were used as controls. Seventy-two hours post-transfection, the localization of PCNP within tumor cells was detected under a fluorescent microscope (Eclipse Ti, Nikon, Melville, NY, USA).

### Reverse transcription-polymerase chain reaction (RT-PCR)

Seventy-two hours post-transfection, total RNA was isolated from the cells using TRIzol reagent, treated with DNase I, and purified using an RNA clean-up kit (Cwbiotech, Beijing, China). Total RNA (1 μg) was applied for cDNA synthesis using a cDNA reverse transcription kit (Cwbiotech, Beijing, China). Primers were designed according to the primer design principles with Primer Premier 5.0 (Premier Biosoft, Palo Alto, CA, USA): PCNP, forward 5’-ATAGGATCCAAAATGGCGGACGGGAAGGCG-3′ and reverse 5′- CCGAAGCTTTTAATTGTCTTGGTCATGGAC-3′; and glyceraldehyde-3-phosphate dehydrogenase (GAPDH), forward 5’-TATGACAACGAATTTGGCTACAG-3′ and reverse 5’-GATGGTACATGACAAGGTGC-3′. The reactions were performed in a total volume of 20 μl using the following thermal cycling parameters: 95 °C for 10 min, 40 cycles of 95 °C for 15 s, 60 °C for 60 s, and 72 °C for 1 min. The results were normalized to the level of GAPDH.

### Cell proliferation and viability assays

The 5-ethynyl-2′-deoxyuridine (EdU) incorporation assay was performed using the Cell-Light EdU Apollo 567 In Vitro Imaging Kit (RiboBio, Guangzhou, Guangdong, China). After incubation with 10 mM EdU for 2 h, SH-SY5Y and SK-N-SH cells were fixed with 4% paraformaldehyde, permeabilized with 0.3% Triton X-100, and stained with fluorescent dyes. 4′, 6-diamidino-2-phenylindole (DAPI) was used to stain the cell nuclei (blue) at a concentration of 5 mg/ml at room temperature for 10 min. Cells were observed under a fluorescent microscope (Eclipse Ti, Nikon, Melville, NY, USA). Cell proliferation rate (%) = (EdU-positive cells)/(total number of cells) × 100 [20]. The cell viability was detected using the CellTiter 96 AQ_ueous_ One Solution Cell Proliferation Assay kit (MTS; Promega, Madison, WI, USA) according to the manufacturer’s protocols.

### Colony formation assay

Cells (4 × 10^2^ per well) were seeded in 6-well plates and cultivated in culture medium at 37 °C for a week. At that point, colonies were washed with phosphate-buffered saline (PBS) buffer for three times before subjected to cell fixation using 1 ml of methanol at room temperature for 15 min. Then, 1 ml of crystal violet was added into each well and incubated for 30 min at room temperature. Plates were gently washed with water and air-dried at room temperature. Finally, the 6-well plate was scanned for colony counting and analysis.

### Wound healing assay

Confluent cells were scratched with a sterile micropipette tip and subsequently washed twice with PBS. The migration distance was photographed under an Olympus CKX41 microscope and then measured using Image J software (National Institute for Health, Bethesda, MD, USA). The migration rate (MR) was calculated as MR (%) = [(A - B)/A] × 100, where A is the width at 0 h, and B is the width at 24 h.

### Soft agar assay

Cells were suspended in 0.6% agarose and medium supplemented with 10% FBS, and the mixture was seeded in 6-well plates containing a basal layer of 1.2% agarose at 1 × 10^4^ cells/well. The medium was replaced twice per week. After 2 weeks of routine culture, colonies were photographed under an Olympus CKX41 microscope. Viable colonies larger than 0.1 mm in diameter were counted.

### Migration and invasion assays

For migration and invasion assays, 1 × 10^5^ cells in serum-free medium were seeded into the upper chamber uncoated or coated with Matrigel (BD Biosciences, San Jose, CA, USA). 500 μl corresponding medium containing 10% FBS was added to the lower chamber. After incubation for 24 h, remaining cells were scrubbed off with cotton swabs, while cells on the bottom surface of the membrane were fixed with 4% paraformaldehyde and stained with 0.1% crystal violet. The cell number was counted using a Zeiss Axioskop 2 plus microscope (Carl Zeiss, Thornwood, NY, USA).

### TdT-mediated dUTP-biotin nick end labeling (TUNEL) assay

TUNEL assay was conducted using an In Situ Cell Death Detection Kit (Beyotime Biotechnology, Shanghai, China) according to the manufacturer’s protocols. Cells were observed under a fluorescent microscope (Eclipse Ti, Nikon, Melville, NY, USA). The percentage of TUNEL-positive cells was calculated using Image J software.

### Western blotting

Seventy-two hours post-transfection, total protein was extracted from SH-SY5Y and SK-N-SH cells. Western blotting was performed to detect the expression of target proteins. The primary antibodies, including anti-extracellular signal-regulated protein kinase 1/2 (ERK1/2), anti-phospho (p)-ERK1/2 (Thr202/Tyr204), anti-c-Jun N-terminal kinase (JNK), anti-p-JNK (Thr183/Tyr185), anti-p38, anti-p-p38 (Thr180/Tyr182), anti-phosphatidylinositol 3-kinase (PI3K), anti-p-PI3K (Tyr458/Tyr199), anti-Akt, anti-p-Akt (Ser473), anti-mammalian target of rapamycin (mTOR), and anti-p-mTOR (Ser2448) antibodies were purchased from Cell Signaling Technology (CST, Danvers, MA, USA). Anti-PCNP, Anti-B-cell lymphoma-2 (Bcl-2), anti-Bcl-2-associated X protein (Bax), anti-B-cell lymphoma-extra large (Bcl-xl), anti-Bcl-xl/Bcl-2-associated death promoter (Bad), anti-cleaved caspase-3, anti-cleaved caspase-8, anti-cleaved caspase-9, anti-Cleaved poly adenosine diphosphate-ribose polymerase (PARP), and anti-GAPDH antibodies were purchased from ProteinTech (Chicago, IL, USA). The horseradish peroxidase-conjugated secondary antibody was purchased from Cell Signaling Technology. The results were normalized to the level of GAPDH. The reaction was visualized using an enhanced chemiluminescence system (Thermo Fisher Scientific, Rockford, IL, USA). The bands were semi-quantified with Image J software.

### Animal study

Animal experiments were approved by the Committee of Medical Ethics and Welfare for Experimental Animals of Henan University School of Medicine (HUSOM-2017-196) in compliance with the Experimental Animal Regulations formulated by the National Science and Technology Commission, China. Animal studies were conducted as previously described with slight modifications [21]. Thirty 4-week-old male BALB/C nude mice (*n* = 6 per group) were obtained from Beijing HFK Bioscience Co., Ltd. (Certificate No. SCXK (Jing) 2014–0004, Beijing, China). SH-SY5Y and SK-N-SH cells (1 × 10^7^ cells in 200 μl PBS) with over-expression and knockdown of PCNP were implanted by subcutaneous injection into the right flanks of mice. The mice were weighed and the tumor volumes were measured daily during the experiment. The tumor volumes were calculated as volume = L × W^2^/2, where L is the longest dimension parallel to the skin surface and W is the dimension perpendicular to L and parallel to the surface [22]. Then the tumor volume doubling time (TVDT) was calculated. TVDT = (T – T_0_) × log2/log(V2/V1), where (T – T_0_) represents the time interval and V2 and V1 indicate the volumes of tumor at the two measurement times [23]. At the end of the experiment, mice were sacrificed and tumors were excised and weighted to evaluate the inhibition rate (IR). The IR of tumor growth was calculated as IR (%) = [(A - B)/A] × 100, where A is the average tumor weight of the control group, and B is that of the treatment group [21].

### Hematoxylin and eosin (HE) staining

After sacrifice, a necropsy examination was immediately performed. Tumor samples were fixed in 10% neutral buffered formalin, embedded in paraffin, sectioned at 5 μm thickness, and processed according to the HE staining protocols. Tumor tissues were observed using a Zeiss Axioskop 2 plus microscope.

### Immunohistochemistry (IHC) and evaluation

Tumor tissues were stained with anti-Ki67 antibody (CST, Danvers, MA, USA), followed by incubation with secondary antibody. Ki67-positive tumor cells were photographed using a Zeiss Axioskop 2 plus microscope and the proliferation index (PI) was determined by the number of Ki67 positive cells among the total number of counted tumor cells [24]. Cluster of differentiation 31 (CD31) has been considered an ideal biomarker for vascular endothelial cells, and its immunostaining density is represented by the tumor microvessel density (MVD) [25]. To determine the tumor MVD, tumor tissues were stained by IHC using CD31 antibody (CST, Danvers, MA, USA). Stained vessels with a clearly defined lumen or well-defined linear vessel shape were observed using a Zeiss Axioskop 2 plus microscope and counted from the representative tumor zone, and the mean value was regarded as MVD.

### Statistical analysis

Data are presented as means ± standard error of the mean. The differences between multiple groups were analyzed by one-way analysis of variance using SPSS 17.0 software, followed by Tukey’s test. A *P* value of less than 0.05 was considered to be statistically significant.

## Results

### Over-expression and down-regulation of PCNP in human neuroblastoma cells

**Fig. 1:**
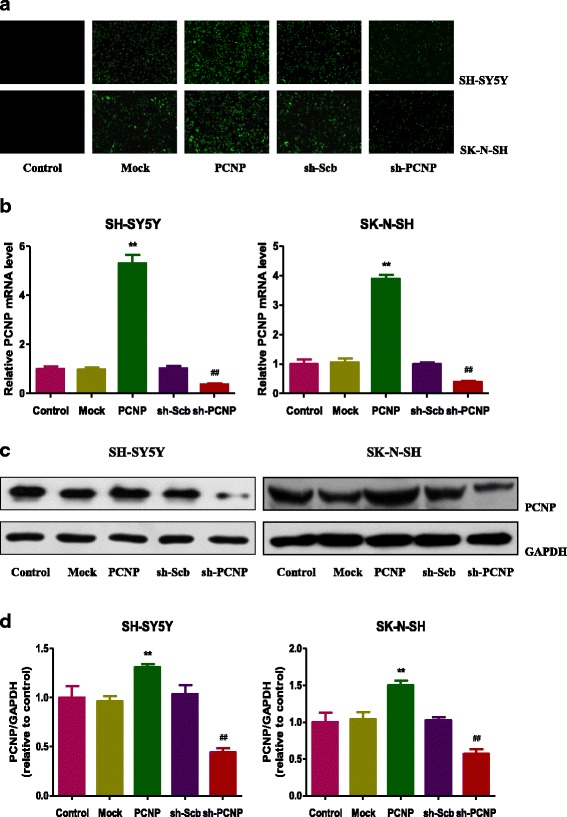
Over-expression and down-regulation of PCNP in human neuroblastoma cells. **a** Fluorescence microscopy of PCNP in SH-SY5Y and SK-N-SH cells; original magnification 100 ×. **b** The expression level of PCNP mRNA was examined by RT-PCR. **c** The protein expression of PCNP was examined by Western blotting. GAPDH was used as the loading control. **d** The densitometry analysis of PCNP was performed, normalized to the corresponding GAPDH level. Data are presented as mean ± SEM of three independent experiments; *P < 0.05, **P < 0.01 compared with the Mock group; ^#^*P* < 0.05, ^##^*P* < 0.01 compared with the sh-Scb group

To investigate the hypothesis that PCNP could influence the growth process of human neuroblastoma cells, PCNP over-expression and knockdown experiments were performed. Transfection of PCNP into SH-SY5Y and SK-N-SH cells resulted in increased nuclear expression of PCNP and transfection of sh-PCNP reduced the nuclear expression of PCNP in SH-SY5Y and SK-N-SH cells (Fig. 1a). In addition, mRNA and protein levels of PCNP showed similar trends (Fig. 1b–d). These results indicate that PCNP over-expression and knockdown experiments have been successfully performed in human neuroblastoma cells.

### PCNP regulates the growth, migration, and invasion of human neuroblastoma cells

**Fig. 2:**
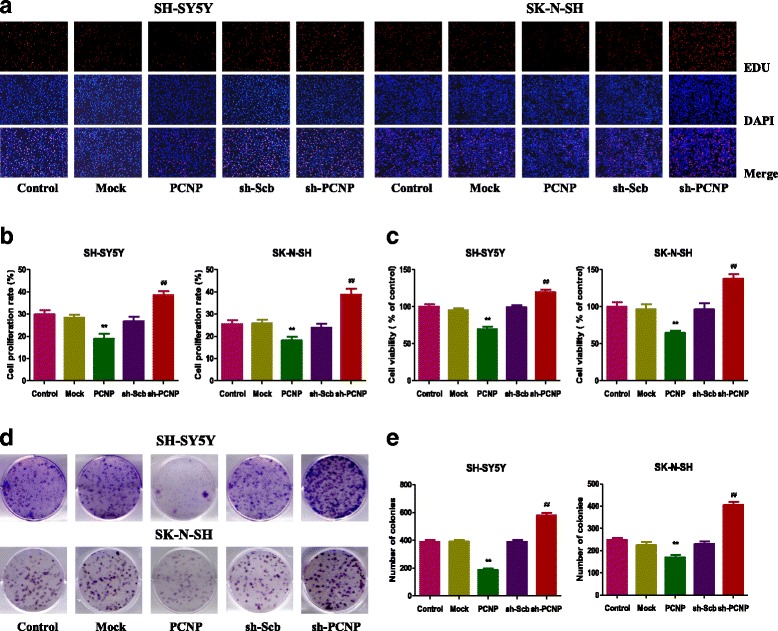
Effects of PCNP on the proliferation and viability of human neuroblastoma cells. **a** DNA replication activities of SH-SY5Y and SK-N-SH cells in each group were examined by EdU assay; original magnification 100 ×. **b** The proliferation rate of each group was analyzed. **c** The percentages of viable cells were determined using MTS assay and the cell viability of the control group was taken as 100%. **d** The clonogenic capacity was determined in SH-SY5Y and SK-N-SH cells. **e** The numbers of colonies were calculated. Data are presented as mean ± SEM of three independent experiments; *P < 0.05, **P < 0.01 compared with the Mock group; ^#^*P* < 0.05, ^##^*P* < 0.01 compared with the sh-Scb group

**Fig. 3:**
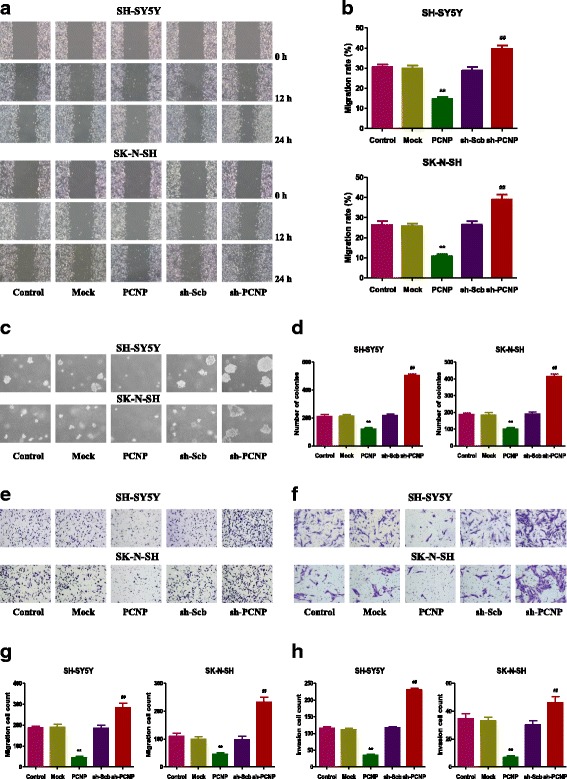
Effects of PCNP on the migration and invasion of human neuroblastoma cells. **a** The effect of PCNP on cell migration was measured by wound healing assay; original magnification 100 ×. **b** Soft agar assay was performed to examine the anchorage-independent survival of cells; original magnification 100 ×. **c** The migration rates of SH-SY5Y and SK-N-SH cells were calculated by the formula shown above. **d** The number of colonies was calculated. **e** Transwell assay was performed to assess the migration of SH-SY5Y and SK-N-SH cells; original magnification 200 ×. **f** Transwell assay was performed to assess the invasion of SH-SY5Y and SK-N-SH cells; original magnification 200 ×. **g** The numbers of the migrated cells were calculated. **h** The numbers of the invasive cells were calculated. Data are presented as mean ± SEM of three independent experiments; *P < 0.05, **P < 0.01 compared with the Mock group; ^#^*P* < 0.05, ^##^*P* < 0.01 compared with the sh-Scb group

As shown in Fig. 2a and b, PCNP over-expression decreased the proliferation of SH-SY5Y and SK-N-SH cells, when compared to the Mock group. In contrast, PCNP knockdown showed opposite effect compared with the sh-Scb group. PCNP showed similar effect on the viability of human neuroblastoma cells (Fig. 2c). Furthermore, over-expression of PCNP reduced the colony formation in SH-SY5Y and SK-N-SH cells and PCNP knockdown markedly increased the number of colonies (Fig. 2d and e). In scratch migration assay, PCNP over-expression attenuated the migration capabilities of SH-SY5Y and SK-N-SH cells and PCNP knockdown exhibited reverse trends (Fig. 3a and b). In soft agar assay, PCNP over-expression attenuated the anchorage-independent growth of SH-SY5Y and SK-N-SH cells and down-regulation of PCNP showed reverse effects (Fig. 3c and d). Transwell analysis showed that neuroblastoma cells transfected with PCNP presented impaired migration and invasion capacities, while the sh-Scb group showed reverse trends (Fig. 3e–h). These results together suggest that PCNP is involved in the growth, migration, and invasion of human neuroblastoma cells.

### PCNP modulates apoptosis of human neuroblastoma cells

**Fig. 4:**
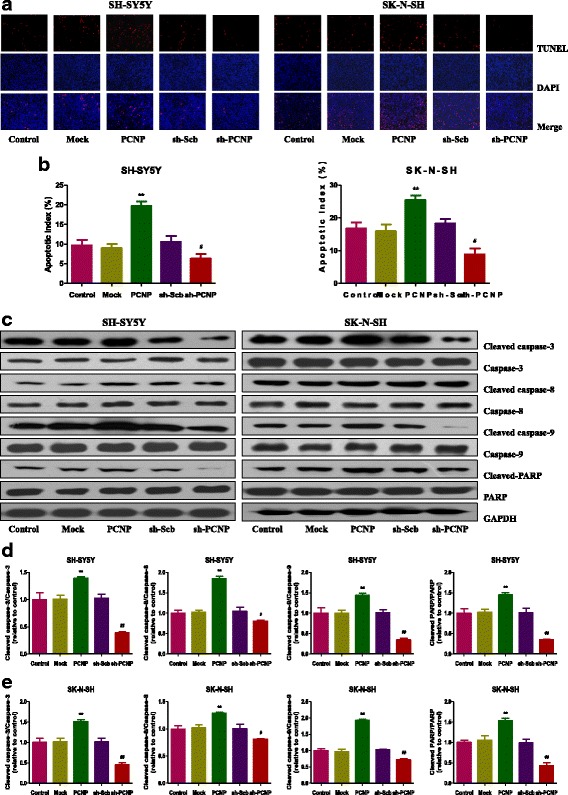
Effects of PCNP on the apoptosis of human neuroblastoma cells. **a** The apoptotic levels of SH-SY5Y and SK-N-SH cells were measured by TUNEL staining; original magnification 100×. **b** The percentages of TUNEL-positive cells were calculated by the formula shown above. **c** Western blotting analysis for the expression of cleaved caspase-3, − 8, and − 9 and cleaved PARP in SH-SY5Y and SK-N-SH cells. GAPDH was used as the loading control. (**d, e**) The densitometry analysis of each factor was performed in SH-SY5Y and SK-N-SH cells, normalized to the corresponding GAPDH level. Data are presented as mean ± SEM of three independent experiments; *P < 0.05, **P < 0.01 compared with the Mock group; ^#^*P* < 0.05, ^##^*P* < 0.01 compared with the sh-Scb group

**Fig. 5:**
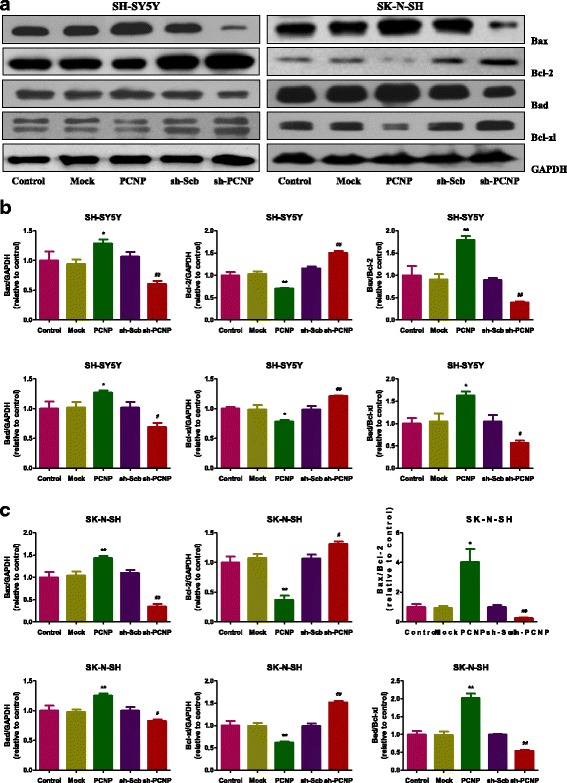
Effects of PCNP on the expressions of Bcl-2 family proteins in human neuroblastoma cells. **a** Western blotting analysis of the expressions of Bax, Bcl-2, Bad, and Bcl-xl in SH-SY5Y and SK-N-SH cells. GAPDH was used as the loading control. (**b, c**) The densitometry analysis of each factor was performed, normalized to the corresponding GAPDH level. The expression ratios of Bax/Bcl-2 and Bad/Bcl-xL were quantified. Data are presented as mean ± SEM of three independent experiments; *P < 0.05, **P < 0.01 compared with the Mock group; ^#^*P* < 0.05, ^##^*P* < 0.01 compared with the sh-Scb group

As shown in Fig. 4a and b, the apoptotic index increased in the PCNP group compared with the Mock group and decreased in the sh-PCNP group compared with the sh-Scb group. The protein expression levels of cleaved caspase-3, 8, 9, and cleaved PARP in human neuroblastoma cells showed similar trends (Fig. 4c–e). The ratio between Bax and Bcl-2 and the ratio between Bad and Bcl-xl have been considered important factors in the regulation of apoptosis. In mammalian cells, increased Bax/Bcl-2 and Bad/Bcl-xl ratios are common phenomena in mitochondrial apoptosis [26, 27]. As shown in Fig. 5, Bax/Bcl-2 and Bad/Bcl-xl ratios increased in the PCNP group compared with the Mock group and decreased in the sh-PCNP group compared with the sh-Scb group.

### PCNP mediates the mitogen-activated protein kinase (MAPK) pathway in human neuroblastoma cells

**Fig. 6:**
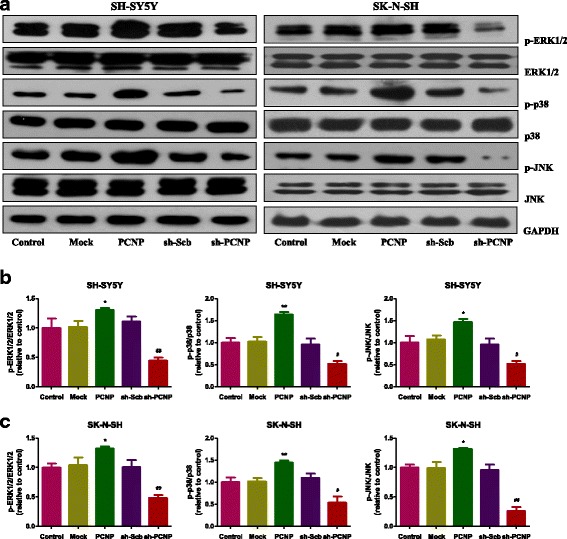
PCNP mediates the MAPK pathway in human neuroblastoma cells. **a** Western blotting analysis of the expressions of ERK1/2, ERK1/2 (Thr202/Tyr204), p38, p38 (Thr180/Tyr182), JNK, and p-JNK (Thr183/Tyr185) in SH-SY5Y and SK-N-SH cells. GAPDH was used as the loading control. **b, c** The intensities of the bands were quantified by densitometry analyses and normalized by the amount of ERK, p38, or JNK. Data are presented as mean ± SEM of three independent experiments; *P < 0.05, **P < 0.01 compared with the Mock group; ^#^*P* < 0.05, ^##^*P* < 0.01 compared with the sh-Scb group

MAPK signaling pathway plays a key role in the regulation of many cellular processes, including cell proliferation, differentiation, and apoptosis [28]. The MAPK family is composed of three major components: ERK1/2, JNK, and p38 protein kinases [29, 30]. As shown in Fig. 6, PCNP over-expression triggered phosphorylations of p38 (Thr180/Tyr182), JNK (Thr183/Tyr185), and ERK1/2 (Thr202/Tyr204) with distinct patterns. However, PCNP knockdown significantly decreased phosphorylations of these protein kinases. The results show that PCNP mediates the MAPK signaling pathway in human neuroblastoma cells.

### PCNP mediates the PI3K/AKT/mTOR pathway in human neuroblastoma cells

**Fig. 7:**
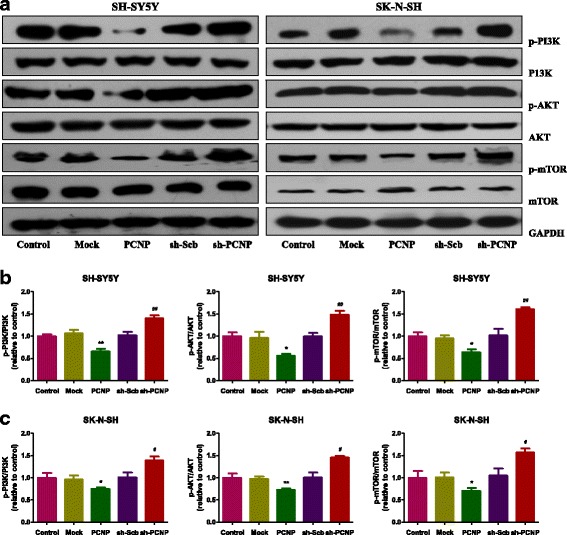
PCNP mediates the PI3K/AKT/mTOR pathway in human neuroblastoma cells. **a** Western blotting analysis of the expressions of PI3K, p-PI3K (Tyr458/Tyr199), AKT, p-AKT (Ser473), mTOR, and p-mTOR (Ser2448) in SH-SY5Y and SK-N-SH cells. GAPDH was used as the loading control. **b, c** The intensities of the bands were quantified by densitometry analyses and normalized by the amount of PI3K, AKT, or mTOR. Data are presented as mean ± SEM of three independent experiments; *P < 0.05, **P < 0.01 compared with the Mock group; ^#^*P* < 0.05, ^##^*P* < 0.01 compared with the sh-Scb group

The PI3K/Akt/mTOR cascade is an important signal transduction pathway involved in most hallmarks of cancer: cell survival, motility, metabolism, and genomic instability [31, 32]. The pathway contributes to cancer-promoting aspects of the tumor environment, including angiogenesis and inflammatory cell recruitment [31]. As shown in Fig. 7, phosphorylations of PI3K (Tyr458/Tyr199), AKT (Ser473), and mTOR (Ser2448) decreased in the PCNP group and increased in the sh-PCNP group, suggesting that PCNP mediates the PI3K/AKT/mTOR signaling pathway in human neuroblastoma cells.

### PCNP regulates the growth and angiogenesis of human neuroblastoma xenograft tumors in nude mice

**Fig. 8:**
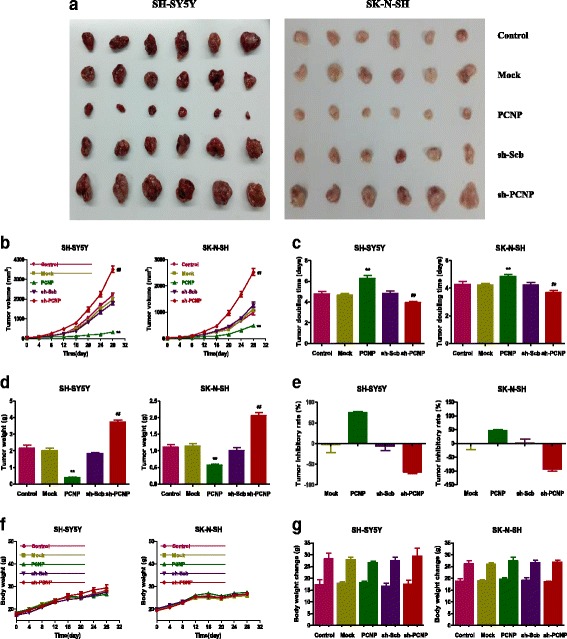
Effects of PCNP on the growth of SH-SY5Y and SK-N-SH xenograft tumors in nude mice. **a** Representative xenografts dissected from different groups of nude mice were shown. (**b, c**) The tumor volume of each group was measured every day and the TVDT was calculated by the formula shown above. (**d, e**) The tumors were weighed and the inhibition rates of tumor growth were calculated by the formula shown above. (**f, g**) The body weight change curve of each group during the experiment and the body weight of each group on the first day (day 0) and the last day (day 28). Values are presented as mean ± SEM (*n* = 6); *P < 0.05, **P < 0.01 compared with the Mock group; ^#^*P* < 0.05, ^##^*P* < 0.01 compared with the sh-Scb group

**Fig. 9:**
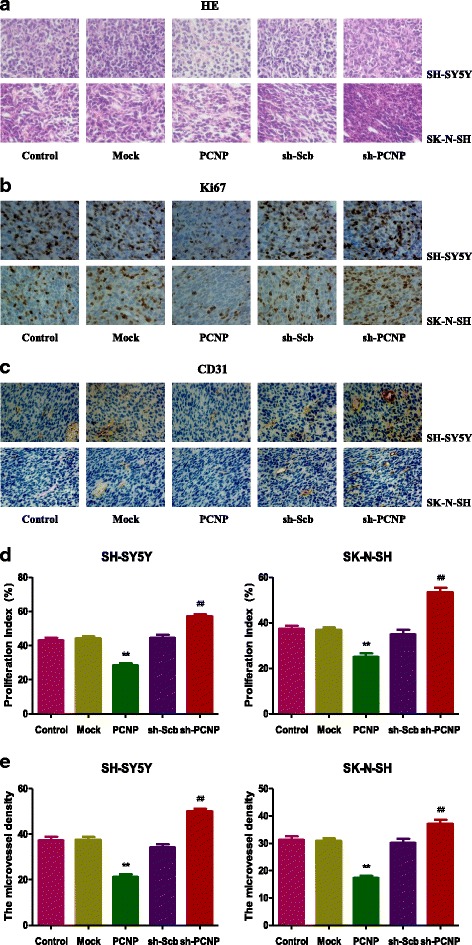
Effects of PCNP on the PI and MVD of human neuroblastoma xenografts. (**a, b, c**) Representive photographs of HE, Ki67, and CD31 staining in SH-SY5Y and SK-N-SH xenograft tumors; original magnification 400×. (**d, e, f**) The PI and MVD were calculated by the formula shown above. Values are presented as mean ± SEM (n = 6); *P < 0.05, **P < 0.01 compared with the Mock group; ^#^*P* < 0.05, ^##^*P* < 0.01 compared with the sh-Scb group

SH-SY5Y and SK-N-SH cells have been widely used to establish subcutaneous xenograft models [33–35]. Then the effect of PCNP on the growth of neuroblastoma xenograft tumors was detected. PCNP over-expression decreased the growth of xenograft tumors, when compared to the Mock group. However, PCNP knockdown increased the growth of xenograft tumors compared with the sh-Scb group (Fig. 8a–e). Furthermore, there was no significant difference in body weight between each group (Fig. 8f and g). IHC with the Ki67 antibody confirmed that the in vivo proliferation of neuroblastoma cells was inhibited in the PCNP group compared with the Mock group and promoted in the sh-PCNP group compared with the sh-Scb group. Moreover, the protein expression of CD31 in neuroblastoma xenograft tumors exhibited a similar trend (Fig. 9). These results together indicate that PCNP could modulate the growth and angiogenesis of human neuroblastoma xenograft tumors.

## Discussion

PCNP is a novel nuclear protein that could interact with NIRF and modulate the transcriptional activity of NIRF [18]. Recent studies suggest that PCNP may play an important role in cell proliferation and tumorigenesis [18, 19]. However, the precise mechanism of action of PCNP in the proliferation, migration, and invasion of cancer cells has not yet been fully elucidated. The human neuroblastoma cell lines SH-SY5Y and SK-N-SH possess several properties of neuronal cells and have been widely used as cellular models to investigate the intracellular mechanisms of action of therapeutic agents [36]. In the present study, SH-SY5Y and SK-N-SH cells were used to evaluate the effects of PCNP in vitro and in vivo. The results demonstrated that the expression of PCNP can be detected in human neuroblastoma cells, in addition to fibrosarcoma cells, hepatoma cells, and myeloid leukemia cells [18]. PCNP over-expression attenuated the proliferation and viability, as well as decreased the migration and invasion capabilities of SH-SY5Y and SK-N-SH cells, whereas PCNP knockdown exhibited completely opposite effects, suggesting that PCNP could play important roles in the growth, migration, and invasion of human neuroblastoma cells.

Apoptosis, also known as programmed cell death, is a critical process for the normal development and maintenance of tissue homeostasis in multicellular organisms [37]. There are two apoptotic signaling pathways: an intrinsic pathway that occurs through the mitochondria and an extrinsic pathway initiated by death receptors [38]. The Bcl-2 family of proteins, such as Bax, Bcl-2, Bad, and Bcl-xl, could function as central regulators of apoptosis in mammals [39]. Caspases could be activated in response to apoptotic stimuli and cleaved caspase-3 could inactivate PARP, thus eventually resulting in the occurrence of apoptotic cascade [40]. Our results showed that PCNP over-expression remarkably increased the apoptotic index, protein expressions of cleaved caspase-3, 8, 9, as well as Bax/Bcl-2 and Bad/Bcl-xl ratios, suggesting the activation of mitochondria-mediated pathway. However, PCNP knockdown dramatically decreased the level of apoptosis, indicating that PCNP has pro-apoptotic function in neuroblastoma.

MAPKs regulate a variety of cellular events, including proliferation, differentiation, and apoptosis, and three major MAPK subfamilies have been identified, ERK 1/2, p38, and JNK [28–30]. Increased expression of p-ERK has been found in many cancers, which can induce cancer cell proliferation and cancer progression [41]. However, many studies have shown that increased p-ERK could promote the apoptosis process in SH-SY5Y cells [42–44]. These controversial results may be attributed to the differences in cell lines [45]. Furthermore, p38 and JNK can be phosphorylated in rotenone-induced apoptosis in SH-SY5Y cells [46]. Our results indicated that PCNP over-expression could induce apoptosis by triggering phosphorylations of p38 (Thr180/Tyr182), JNK (Thr183/Tyr185), and ERK1/2 (Thr202/Tyr204) in both SH-SY5Y and SK-N-SH cells. However, PCNP knockdown could promote the growth, migration, and invasion of neuroblastoma cells by decreasing phosphorylations of p38 (Thr180/Tyr182), JNK (Thr183/Tyr185), and ERK1/2 (Thr202/Tyr204). These results together suggest that PCNP can regulate the growth process of human neuroblastoma cells via the MAPK signaling pathway.

The PI3K/Akt/mTOR signaling pathway plays important roles in promoting cell survival, growth, motility, and protein synthesis [32, 47, 48]. PI3K activates the serine/threonine kinase Akt, which in turn phosphorylates and activates mTOR through a cascade of regulators [47]. Activation of the PI3K/AKT/mTOR pathway is involved in tumor progression and reduced patient survival [49]. It has been widely accepted that PI3K/AKT/mTOR pathway is a promising therapeutic target for the treatment of cancer [32, 47, 50]. A recent study indicates that alectinib could suppress cell proliferation and induce apoptosis through the inhibition of PI3K/Akt/mTOR signaling in neuroblastoma cells [51]. Moreover, afatinib exhibits anti-tumor efficacy by inducing apoptosis and blocking the PI3K/AKT/mTOR signaling in a neuroblastoma xenograft mouse model [52]. Similarly, our results showed that PCNP over-expression significantly induced apoptosis by inhibiting phosphorylations of PI3K (Tyr458/Tyr199), AKT (Ser473), and mTOR (Ser2448), suggesting that PCNP-associated agents can be developed as anti-cancer drugs. Nevertheless, PCNP knockdown promoted the growth, migration, and invasion of neuroblastoma cells via increasing phosphorylations of PI3K (Tyr458/Tyr199), AKT (Ser473), and mTOR (Ser2448). These data reveal that PCNP can regulate the growth, migration, and invasion of human neuroblastoma cells through the PI3K/Akt/mTOR signaling pathway.

A number of studies indicate that SH-SY5Y and SK-N-SH cells have been widely adopted to establish subcutaneous xenograft models [33–35]. We therefore examined the effect of PCNP on the growth of neuroblastoma xenograft tumors in BALB/c nude mice. PCNP over-expression significantly decreased the growth of neuroblastoma xenograft tumors, whereas PCNP knockdown notably promoted tumor growth. However, the tumor inhibitory rate of PCNP in SH-SY5Y cells was higher than that in SK-N-SH cells, which can be attributed to the difference of the level of GD2 ganglioside expression between SH-SY5Y and SK-N-SH cells [53, 54]. Ki67, a nuclear non-histone protein, can be detected in proliferating cells in all stages of the cell cycle except G0 [55]. The expression of Ki67 closely associates with the proliferation, invasiveness, and clinical outcome of a variety of malignant tumors [56]. Ki67 is considered an important marker and has been widely used in detecting the proliferation of malignant cells [23, 55, 56]. The results showed that the expression of Ki67 was decreased in the PCNP group and increased in the sh-PCNP group, which was in good agreement with the above findings. CD31 is an ideal biomarker for vascular endothelial cells and its density is represented by the tumor MVD [24, 25]. The results indicated that PCNP over-expression reduced the expression of CD31, while PCNP knockdown promoted the expression of CD31 in neuroblastoma xenograft tumors, suggesting that PCNP could modulate the growth of human neuroblastoma xenograft tumors by regulating angiogenesis.

## Conclusions

We demonstrate that the expression of PCNP can be detected in human neuroblastoma cells. The study suggests that PCNP could mediate the proliferation, migration, and invasion of human neuroblastoma cells through MAPK and PI3K/AKT/mTOR signaling pathways. Considering its importance in the developmental process of human neuroblastoma cells, PCNP could be a potential therapeutic target for advanced and recurrent human neuroblastoma. The finding that over-expression of PCNP reduces the growth of human neuroblastoma xenograft tumors by regulating angiogenesis makes PCNP a potential therapeutic target.

## Abbreviations

Bad: Bcl-xl/Bcl-2-associated death promoter
Bax: Bcl-2-associated X protein
Bcl-2: B-cell lymphoma-2
Bcl-xl: B-cell lymphoma-extra large
CD31: Cluster of differentiation 31
cDNA: Complementary deoxyribonucleic acid
DAPI: 4′, 6-diamidino-2-phenylindole
E: Glutamic acid
EdU: 5-ethynyl-2′-deoxyuridine
ERK1/2: extracellular signal-regulated protein kinase 1/2
FBS: Fetal bovine serum
GAPDH: Glyceraldehyde-3-phosphate dehydrogenase
HE: Hematoxylin and eosin
IHC: Immunohistochemistry
IR: Inhibition rate
JNK: c-Jun N-terminal kinase
MAPK: Mitogen-activated protein kinase
MR: Migration rate
mTOR: mammalian target of rapamycin
MVD: Microvessel density
NIRF: Np95/ICBP90-like RING finger protein
P: Proline
PARP: Poly adenosine diphosphate-ribose polymerase
PBS: Phosphate-buffered saline
PCNP: PEST-containing nuclear protein
PI: Proliferation index
PI3K: phosphatidylinositol 3-kinase
RT-PCR: Reverse transcription-polymerase chain reaction
S: Serine
shRNA: Short hairpin ribonucleic acid
T: Threonine
TUNEL: TdT-mediated dUTP-biotin nick end labeling
TVDT: Tumor volume doubling time
